# Evaluating the potential role of pleiotropy in Mendelian randomization studies

**DOI:** 10.1093/hmg/ddy163

**Published:** 2018-05-16

**Authors:** Gibran Hemani, Jack Bowden, George Davey Smith

**Affiliations:** MRC Integrative Epidemiology Unit, Population Health Sciences, University of Bristol

## Abstract

Pleiotropy, the phenomenon of a single genetic variant influencing multiple traits, is likely widespread in the human genome. If pleiotropy arises because the single nucleotide polymorphism (SNP) influences one trait, which in turn influences another (‘vertical pleiotropy’), then Mendelian randomization (MR) can be used to estimate the causal influence between the traits. Of prime focus among the many limitations to MR is the unprovable assumption that apparent pleiotropic associations are mediated by the exposure (i.e. reflect vertical pleiotropy), and do not arise due to SNPs influencing the two traits through independent pathways (‘horizontal pleiotropy’). The burgeoning treasure trove of genetic associations yielded through genome wide association studies makes for a tantalizing prospect of phenome-wide causal inference. Recent years have seen substantial attention devoted to the problem of horizontal pleiotropy, and in this review we outline how newly developed methods can be used together to improve the reliability of MR.

## Introduction

Of fundamental importance to medical and social sciences is being able to elucidate how one phenotype (the exposure) causally relates to another (the outcome). Mendelian randomization (MR) is a method that strengthens causal inference by using natural genetic variation to mimic a randomized controlled trial (RCT) (1,2) [see Appendix 1 for a brief recap of the method and its assumptions; for readers not familiar with Mendelian randomization reading the current paper in conjunction with Davey Smith and Hemani (2) is recommended]. MR unlocks the potential to exploit the massive wealth of genetic associations (3) accrued through over a decade of genome-wide association studies (GWAS) (4) for causal inference, but the method is not a panacea. As such, the four years since our earlier review in HMG (2) has seen considerable developments of methods aimed at improving the reliability and scope of MR, and a concomitant explosion in the use of MR across a broad range of disciplines (5). We have also seen the emergence of genotyped biobank data that contribute to the ever-growing sample sizes of GWAS (6), and herald a commitment from governments to population scale genetic studies. Consequently, the breadth and manner in which MR is performed has shifted quite dramatically.

Particularly impactful has been growth in the use of GWAS summary data (5,7) (see Box 1). Here, causal inference can be made using data from only the summary estimates of GWAS, leading to a number of strategic advantages (8). First, these summary associations (which constitute ‘the data’) are non-disclosive, and often freely and publicly available for potentially thousands of traits. This enables high throughput automation simply by recycling existing results. Second, the genome is used as an anchor between traits, allowing causal inference to be made for pairs of traits that may never have been recorded in the same samples. This dramatically enlarges the space of possible causal inference tests. Third, statistical power issues are ameliorated by harnessing the massive sample sizes in GWAS, which are each individually conducted to maximize the power for a particular trait.

Box 1. The data required for MR analyses

 In the simplest case all that is required to perform MR is knowledge of the SNP-exposure association(s) (effect size and standard error) and the SNP-outcome association(s) [effect size and standard error, with the effect size relative to the same effect allele as the SNP-exposure association(s)]. A data frame of SNP-exposure and SNP-outcome association results is termed a *summary set* (72).
 These data can be obtained simply from published summary data from genome-wide association studies, which are often freely available and non-disclosive about study participants. Typically, the instruments for an exposure are readily available through publications, the GWAS catalog (3), or other resources (20,67,102) in which reliable and reproducible associations are reported. The corresponding SNP-outcome associations are harder to identify because they are unlikely to be GWAS significant and therefore typically of less interest in the primary GWAS publication. MR-Base (8) and PhenoScanner (101) are two resources that now provide searchable databases comprising complete GWAS summary data (i.e. results from all the SNPs tested, not just those that were significant).
 MR analyses that use only summary data have been called summary MR (SMR) (30) and two-sample MR (2SMR) (7). But there are actually more accurate ways to categorize the data contexts for MR analyses.
• **Individual level data**—here the SNPs, exposure phenotype and outcome phenotype are all measured in the same sample. Ideally the SNPs that are to be used as instruments have been identified from an external source. Individual level data are valuable because it can be used to perform some sensitivity analyses that cannot be done with summary data, e.g. the use of interactions (87,90,91), and triangulating MR estimates with alternative causal inference strategies (16,18,103). Other advantages of using individual level data from the same sample are that causal estimates are robust to misspecification of the SNP-exposure association model, and when LD patterns are needed an external reference panel can be avoided. These are not true for two-sample approaches (54).
• **One-sample using summary data**—here summary data are available for the relevant SNPs for the exposure and outcome traits, however the data used to generate these two datasets came from the same samples. If the instruments are weak then the residual variance between the exposure and outcome effect estimates will have shared correlation structures, which means that they could be biased in the direction of the observational estimate. The same applies to individual level data.
• **Two-sample using summary data**—here the summary data for the exposure is generated from an entirely different set of samples from those used to obtain the outcome summary data. Because the uncertainty in the SNP-exposure and SNP-outcome association estimates is independent, weak instrument bias will be in the direction of the null. If, however, there is partial overlap between the exposure and outcome samples, then the bias will tend in the direction of the null or the observational estimate depending on the proportion of overlap (7).

While MR offers an attractive solution to causal inference using observational or non-interventional data, it essentially replaces traditional epidemiological assumptions (9) with other assumptions (Appendix 1 and Table 1). A number of reviews have appeared recently that relate to the scope of MR (10,11), emerging methods (11,12), applications to drug discovery (13–15), and comparisons to other causal methods (16–18). The limitations are numerous (for extensive discussion, see 1,8), and much focus of methodological development in the past few years has been on the problem of pleiotropy (Box 3). To this end, the objective of this review is to contextualize recent methods and to provide insight into how they can be used in conjunction with one another to interrogate and ameliorate issues surrounding pleiotropy in MR (Table 2).

**Table 1.** Assumptions in 2SMR adapted from ref. (56) and expressions based on variable definitions in Appendix 1

Assumption	Description
***General IV assumptions***	
**IV1**	$\gamma_{j}>0$, the SNP predicts the exposure
**IV2**	$k_{x}\psi_{j}=0,\;k_{y}\psi_{j}=0$, there is no SNP-confounder association
**IV3**	$\alpha_{j}=0$, the SNP does not exhibit horizontal pleiotropy
***2SMR assumptions***	
**2SMR1**	The causal relationship is identical in the two samples
**2SMR2**	$cov\left(\varepsilon_{x_{j}},\varepsilon_{y_{j}}\right)=0$
**2SMR3**	The error variances are known
***No measurement error in the exposure (NOME)***	
	$var\left(\varepsilon_{x_{j}}\right)\approx 0$, the SNP-exposure effect is estimated with negligible error
***Instrument Strength Independent of Direct Effect (InSIDE)***	
	$cov\left(\gamma_{j},\;\alpha_{j}\right)=0$

**Table 2.** Strategies for combining different MR methods in different contexts

Strategy	Description	Limitations
***A. Single-instrument MR, for a single hypothesis or hypothesis-free scan***		
Genetic colocalization +Bi-directional MR +MR Steiger test +Mediation-based analysis	Use genetic colocalization to eliminate possibility distinct causal variants (25,30,31); if instruments are available for the outcome then test the reverse causal effect (110); if not use MR Steiger (43); use genetic mediation-based analysis (40,111) to try to separate horizontal and vertical pleiotropy	Statistical power may be low, and MR methods cannot separate horizontal from vertical pleiotropy. Genetic mediation-based methods are susceptible to measurement error and confounding, and require individual level data. MR-RAPS requires instrument selection, SNP-exposure effect estimation and SNP-outcome effect estimation from independent samples
***B. Single hypothesis analysis with multiple instruments***		
IVW random effects or MR-RAPS +Heterogeneity tests +MR-Egger, weighted median, weighted mode +Leave-one-out analysis +Negative controls	Begin with simplest model and then test for heterogeneity; if heterogeneity is present then perform sensitivity analyses	Power of heterogeneity test is low; this is not a principled way to decide the reliability of the result; use of negative control samples requires individual level data and availability of an appropriate GxE or GxG interaction
Rucker framework	Use Q and Q’ heterogeneity statistics to navigate between 4 different models of horizontal pleiotropy	Restricted to specific models of horizontal pleiotropy, and statistical power drops substantially when pleiotropic model increases in complexity
Bayesian model averaging	Average across 3 different models of horizontal pleiotropy	As above; difficult to make decision if the posterior distribution is multi-modal
***C. Hypothesis-free analysis of exposure with multiple instruments***		
IVW random effects or MR-RAPS Follow up using section B	Use single method to identify putative associations, then follow up with a strategy from section B	Highest power but likely also highest false discovery rate; MR-RAPS requires that exposure and outcome has no sample overlap which can be difficult to prove
Weighted mode estimate	Use single method for all tests, simulations suggest highest performance in terms of high power and low FDR for a single method. Follow up with a strategy from section B	Bandwidth parameter cannot be estimated
MR-MoE	Use machine learning approach to select the estimate for each test. Follow up with a strategy from section B	Potentially slower to run, does not give information regarding why a particular method was chosen

## The Single Instrument Case

Suppose we have a single genetic instrument for the exposure. This is a common scenario especially for ‘omic’ variables, such as gene expression (19), DNA methylation (20) and protein levels (21) where there is typically a strong genetic association nearby the genomic location of the variable, typically referred to as a *cis*-effect (22). An estimate of the causal effect can be obtained from a Wald ratio: the influence of the SNP-outcome effect divided by the SNP-exposure effect (23) (Appendix 1). A qualitative inference as to whether the exposure is causally related to the outcome is most simply obtained by testing if the instrumenting SNP associates with the outcome. This result is only reliable, however, if the SNP-outcome association is due to vertical pleiotropy through the exposure (see Box 2). Alternatively, it could arise due to horizontal pleiotropy, where the SNP influences the exposure and outcome through independent pathways, or *distinct causal variants* (24) where the SNP that influences the exposure is in linkage disequilibrium (LD) with another SNP that independently influences the outcome. Evaluating the possibility of distinct causal variants can be achieved through the use of genetic colocalization methods (25)—those that attempt to evaluate if two traits share the same causal variant at a particular locus. While not *sufficient*, shared causal variants between two traits are *necessary* for them to be causally related. Thus, the use of co-localization in MR can be valuable to eliminate at least some unreliable associations.

Box 2. Pleiotropy in the MR context

**Figure 1.**
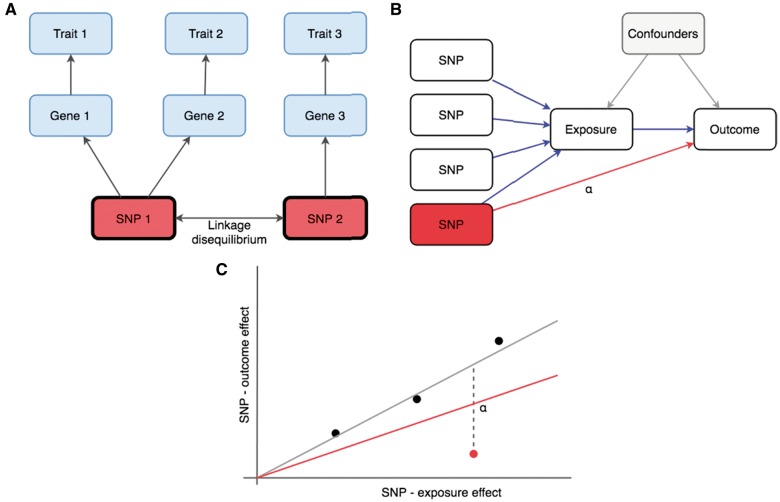
** (A)** The same SNP can associate with multiple traits due to vertical pleiotropy, horizontal pleiotropy and linkage disequilibrium with distinct causal variants depending on the analytical context. To estimate the causal influence of gene expression level (Gene) 1 on Trait 1, SNP 1 is a valid instrument that acts in a vertical pleiotropic manner. But SNP 1 has a horizontal pleiotropic effect when using it to estimate the causal influence of Gene 1 on Trait 2. If SNP 1 was used to instrument Gene 1 to test its effect on Trait 3, it would exhibit a pleiotropic association through linkage disequilibrium with SNP 2. **(B)** A directed acyclic graph (DAG) in which four SNPs instrument an exposure. The fourth SNP has a horizontal pleiotropic effect of magnitude α. The impact of the horizontal pleiotropic effect is shown in the scatter plot in **(C)**, where the grey slope represents the true causal effect obtained from the three valid instruments, and the red slope represents the IVW estimate when all SNPs are used as instruments.

- The human phenome can be described as all (measurable or not) characteristics of an individual (104). While inherited natural genetic variation is largely uniform across tissues and over time (barring somatic mutations, etc.), natural phenotypic variation is massively multi-dimensional and dwarfs the genome in scale and complexity.
- Given that the majority of measurable phenotypes have a heritable component (105), pleiotropy in the most general sense—the phenomenon of a single genetic variant influencing multiple traits—must be very common.
- From a statistical perspective, MR returns a ‘positive result’ if a SNP known to influence the hypothesized exposure also influences the hypothesized outcome. This is the precise definition of pleiotropy. In the interests of reliable causal inference, what is of crucial importance is divining the mode of pleiotropic action: does the SNP influence the outcome because the exposure influences the outcome? This is the mechanism assumed in MR and will be referred to as vertical pleiotropy in this review, but it has also been termed mediated pleiotropy (106), type II pleiotropy (107), secondary pleiotropy (108), spurious pleiotropy (109), and in some literature it is not considered to be pleiotropy at all.
- There are two alternative mechanisms by which a SNP could associate with two phenotypes. First, a SNP could influence the outcome through a pathway other than the exposure. In this review, we will refer to such an effect as horizontal pleiotropy, though it has also been called biological pleiotropy (106), Type I pleiotropy (107), developmental pleiotropy and selectional pleiotropy (73). The second alternative mechanism is through distinct causal variants (24), where a SNP exhibits a statistical association with two traits simply because it causally relates to one trait while also being in LD with a causal variant for another trait.
- Within the context of a single MR analysis (i.e. one exposure on one outcome) a single genetic variant could simultaneously exhibit different modes of pleiotropy (Fig. 1).

Several colocalization methods are now widely used (24,26–31). The R/coloc (25) package uses summary data for the SNPs in a region and estimates the posterior probability of shared genetic factors by evaluating the similarity of effect size patterns across the region. The joint likelihood mapping (JLIM) approach (31) adopts a similar tactic but also requires that the LD pattern between the SNPs in a region for one of the two traits is available. The heterogeneity in dependent instruments (HEIDI) approach (30) is slightly more flexible—it is another form of colocalization analysis using LD information but is typically applied using an external reference panel in which the effect sizes are estimated in different samples from the LD patterns. S-PrediXcan (32) adopts a similar strategy of using an LD reference panel with summary data for genetic colocalization.

There are two important factors that can lead to inaccuracies in these methods. First, if there are multiple conditionally independent causal variants (33–35) in the *cis* region, as is often reported (19,20,36), then this could lead to incorrectly declaring shared causal variants. Using the methods in conjunction with conditional analysis is recommended to mitigate this problem (25,30). Second, if the exposure and outcome trait effects were estimated in populations with different LD patterns then the patterns of effect sizes may not correspond according to the underlying genetic architecture. This problem is difficult to overcome, and ideally one would demonstrate replication in independent samples.

While reliable colocalization results can eliminate distinct causal variants as a potential explanation for a strong SNP-outcome association, vertical pleiotropy in the single instrument case is impossible to prove using summary data for two traits alone (36). Triangulation, the practice of evaluating the same question using different methods (16,18) that have non-overlapping limitations, must be applied in this scenario. Genetic mediation-based analyses (37–40) are more liable to problems of confounding and measurement error than MR (41–43), but could potentially separate between vertical and horizontal pleiotropy in some scenarios. Network construction to evaluate consistency of effects (an alternative form of mediation analysis using MR) can also be used (44).

## Causal Inference Using Multiple Genetic Variants

Many complex traits for which GWAS has been performed using very large sample sizes return tens or hundreds of independent genetic variants reaching the established genome wide significance level (4). Independence is often ensured using LD-based clumping and pruning (45). In these cases, extending the analogy to RCTs, each instrumenting SNP is considered an independent experiment (in the sense that they independently modify the exposure), and as such the results from each experiment can be meta-analysed to give an overall estimate (7,46,47). Most simply, a fixed effects inverse variance weighted (IVW) meta-analysis method is used, where the contribution of each SNP to the overall estimate is the inverse of the variance of its effect on the outcome (See Box 3).

Box 3. Weights used in IVW analysis

- When multiple SNPs are available as instruments for a particular analysis, the causal estimates from each SNP can be meta-analysed (or averaged) to yield a more precise estimate. The weights used in the standard inverse variance weighted (IVW) meta-analysis (first-order weights), make two assumptions. Firstly, that the SNP-exposure and SNP-outcome association estimates are uncorrelated (so that covariance terms can be ignored) and secondly that the SNP-exposure association is measured with infinite precision [the NO Measurement Error in the exposure (NOME) assumption]. In practice the NOME assumption is always violated because the SNP-exposure association standard errors are always non-zero, but when the NOME assumption is strongly violated (as measured by a small average F-statistic across the SNPs), the IVW estimate will suffer from regression dilution bias towards the null. The magnitude of this dilution is inversely proportional to F. First-order weights are also traditionally used by Cochran’s Q statistic to test for the presence of heterogeneity, which is used to infer the presence of horizontal pleiotropy in MR. In this case, strong NOME violation leads to Cohran’s Q detecting heterogeneity too often when in fact no pleiotropy is present (56) (i.e. type I error rate inflation).
- So-called second-order weights, which combine the Wald ratio estimates and the standard errors for the SNP-exposure and SNP-outcome estimates, attempt to ameliorate the problem of NOME violation but in fact produce IVW estimates that suffer from even stronger regression dilution bias than first-order weights. They also dramatically reduce the power of Cochran’s Q statistic to detect heterogeneity due to pleiotropy when it is truly present (51) (i.e. type II error rate inflation). Incorporating Modified second-order weights into the IVW estimate and Cochran’s Q statistic has been shown to correctly address both issues, removing the effect of regression dilution bias and furnishing Q statistics with the correct operating characteristics (51). Modified second-order weights are also incorporated into the MR-RAPS (54) estimator. A simulation-based heterogeneity and outlier test is proposed within MR-PRESSO (61), which performs similarly to modified second-order weighting of Cohran’s Q statistic.

There are two major advantages that arise when multiple instruments are available. First, the statistical power potentially improves, which is particularly important because each SNP-outcome association on its own is typically small. Second, the problem of horizontal pleiotropy can begin to be addressed. One important extension of IVW analysis is the weighted generalized linear regression method (47). Here, the SNPs used as instruments can be correlated, as in the case of multiple conditionally independent variants acting in *cis* on a gene expression level. A reference LD panel is used to account for the correlation structure thus avoiding ‘double counting’ of SNP effects.

If the exposure influences the outcome and the SNPs only directly influence the exposure, we expect that the influence of each SNP on the outcome is proportional to the effect of the SNP on the exposure. This proportional factor (the causal effect) will be the same across SNPs, making their individual causal ratio estimates homogeneous. The more SNPs that satisfy this expectation, the less likely it is that the SNP-outcome associations are arising simply because of horizontal pleiotropy (or distinct causal variants) (48). It is important to note that the proportionality of SNP-exposure and SNP-outcome effects could arise due to *perfect confounding*—where all the SNP-exposure instruments actually arise due to another trait influencing both the exposure and the outcome.

Invariably we know we can test whether the instrumenting SNPs associate with the outcome, but inferring why that association is present is difficult. Much of the recent method development in MR, which we will now go on to describe, has focused on modelling the Wald ratios from multiple instrumenting SNPs in an attempt to separate the vertical pleiotropic pathway (i.e. the hypothesized causal pathway) from any other influences.

## Testing for Heterogeneity to Gauge the Problem of Pleiotropy

Because the IVW estimate is essentially a weighted average of the Wald ratios obtained from each SNP, if any of the SNPs exhibit horizontal pleiotropy (i.e. influencing the outcome through a pathway other than the exposure) then the causal effect estimate is liable to be biased. Thus, in principle the IVW estimate is said to have a 0% ‘breakdown level’ because it is not guaranteed to tolerate any SNPs violating the third IV assumption (exclusion restriction assumption). A tool used extensively in meta-analysis is to assess the heterogeneity between studies is Cochran’s Q statistic (49), and it can also be applied in the MR context (50,51). Here, substantial heterogeneity among the Wald ratios for each SNP could indicate a variety of potential problems, most notably that at least one (but possibly several or even all) of the SNPs is exhibiting horizontal pleiotropy.

Though not the subject of this review it is important to note that there are many other factors that could induce heterogeneity among the causal ratio estimates of a set of SNPs, in the total absence of pleiotropy. For example, heterogeneity could arise because (but not limited to):

- The outcome of interest is a binary variable (e.g. a disease status), and the SNP-outcome associations are measured on the odds ratio scale. Heterogeneity in this case is due to the non-collapsibility of the odds ratio as a summary measure, meaning that each SNP is estimating a slightly different causal parameter (52);
- The samples used to estimate the SNP-exposure and SNP-outcome associations are not homogeneous e.g. a difference in the distribution of a covariate confounding the exposure-outcome relationship across samples could induce heterogeneity (54);
- The SNP-exposure and SNP-outcome relationships are not correctly specified—i.e. in the two-sample setting the causal relationship between the exposure and the outcome is different in each of the samples (53,54).

Heterogeneity is therefore a sign that either the modelling assumptions are wrong, or the IV assumptions are violated.

### Balanced horizontal pleiotropy

Suppose that all the SNPs exhibit horizontal pleiotropy, such that each SNP influences both the exposure and also the outcome through another pathway. In this scenario, we can model the SNP-outcome effect as being the influence of the SNP on the outcome through the exposure, but in addition each SNP is also allowed a random positive or negative effect on the outcome through some other pathway. Here, it is assumed that on an average the random effects have zero mean and are uncorrelated with the SNP-exposure effect (55) (Appendix 1). In this instance the overall IVW estimate is asymptotically unbiased as the number of SNPs grows large, and the correct standard error can be obtained from fitting a random effects IVW model (56).

While there is often concern that horizontal pleiotropy will induce false positive causal associations, it can also reduce the true positive rate. In the universal pleiotropy model described earlier, the horizontal pleiotropy introduces noise to the causal association which means that statistical power will be reduced.

### Directional (unbalanced) horizontal pleiotropy

In the case of balanced horizontal pleiotropy, it is assumed that the random effects have zero mean, which will lead to the IVW estimate being unbiased. However, an alternative possibility is that the random effect does not have zero mean, and that the average random effect is *directional*. In this scenario, the IVW estimate will be biased.

A simple approach to account for this bias is to use MR-Egger regression (55,57), which differs from the IVW estimate by allowing a non-zero intercept. The intercept term represents an estimate of the directional pleiotropic effect. In an analysis of the causal influence of serum urate levels on coronary heart disease (CHD) it was shown that a strong positive relationship returned by the IVW estimate was almost entirely nullified after accounting for directional pleiotropy in the MR-Egger model (58).

There are three important factors to consider when using standard MR-Egger regression. First, it is required that the SNP-exposure estimates are oriented to be positive, and the SNP-outcome effects are flipped accordingly. This is done so that the SNP-exposure association reflects the `weight’ it receives in the analysis. The need to perform re-orientation has recently been relaxed with a modification of the original MR-Egger model based on Radial regression (59). Second, the statistical power of MR-Egger analysis is dramatically lower than IVW analysis, particularly when the SNP-exposure effect sizes are relatively homogeneous (56). Third, such homogeneity also means that MR-Egger analyses are more susceptible to regression dilution bias (57,60). Simulation extrapolation (SIMEX) corrections can be applied to account for regression dilution bias (57).

Finally, both the IVW and MR-Egger frameworks are dependent on the so-called InSIDE assumption (Instrument Strength Independent of Direct Effect). This justifies treating pleiotropy as a random effect. Furthermore, the MR-Egger assumptions are in fact a subset of the IVW assumptions, because the former relaxes the additional assumption that the average pleiotropic effect is zero. If the InSIDE assumption is violated and the SNP-exposure effects are correlated with the horizontal pleiotropic effects, then bias will be incurred. InSIDE violation is very likely when a sizable proportion of the horizontal pleiotropy operates through a confounder of the exposure-outcome relationship (56).

## Outlier Removal

Random effects IVW and MR-Egger analyses relax the exclusion restriction assumption, specifically in the special cases described above where all the SNPs are allowed to exhibit a random horizontal pleiotropic effect and thus the methods have a *maximum breakdown level* of 100% (i.e. remains asymptotically unbiased even when all SNPs exhibit horizontal pleiotropy. However, these methods are liable to bias under many other patterns of horizontal pleiotropy.

Several methods now exist that operate on the model that only some proportion of the SNPs will have a horizontal pleiotropic effect. They attempt to reduce heterogeneity by removing SNPs that contribute to the heterogeneity disproportionately more than expected given the standard errors of the Wald ratios. Such outlier removal strategies are present in the MR-PRESSO (61), and generalized summary MR (GSMR) approaches (62). Cochran’s Q statistic has also been extended to enable more reliable outlier detection, especially with weak and pleiotropic genetic instruments (51). The detection of outliers is also automated in the Radial MR framework (59).

### Down-weighting outliers

While IVW and MR-Egger use a mean-based approach to obtain an overall estimate, one way to avoid the contribution of some invalid instruments is to instead base the overall estimate on the median of the instruments (63,64). Here, it is assumed that at least 50% of the instruments are valid. This can be extended to a more efficient weighted analysis, which then requires that the set of instruments accounting for 50% or more of the total weight is valid (64).

A further variation is to employ the zero modal pleiotropy assumption (ZEMPA) and calculate the weighted mode of the Wald ratio estimates (65). The majority of the SNPs could be invalid (and hence the median unreliable), but providing the set of SNPs which form the largest homogeneous cluster are valid, the modal Wald ratio will be asymptotically unbiased. Some decision-making is required of the user in this scenario, because in order to obtain the clustering of effects it is necessary to choose a bandwidth. It is prudent to perform sensitivity analyses that evaluate the consistency of the overall estimate using different bandwidths.

### Reasons to be wary of outlier adjustment

Median and mode-based estimators can be viewed as implicit outlier removal approaches, since they only allow the SNPs in the majority to contribute to the overall estimate. Using a weighting approach may help to mitigate some of the issues that arise from explicit outlier removal, e.g. in the ‘omic setting described earlier, a single *cis*-acting variant might account for >50% of the weight even when there are many SNP effects from elsewhere in the genome (*trans*-effects) (20,66,67).

One issue with outlier removal (or down-weighting) is that it is at some level a form of cherry picking—generally the standard error of the causal effect estimate will be reduced after removing those SNPs that appear to deviate from the majority. There are also good examples where the SNP that might appear to be the outlier is in fact the most biologically reliable. For example, for ‘omic variables where there are potentially many *trans*-effects but only one *cis*-effect, the *cis*-effect is likely closer to the biology of the molecular trait due to its genomic proximity. By contrast in order for the *trans* SNP to exert an influence on the molecular trait it is presumed that it must go through several pathways, opening the possibility that those pathways influence the outcome independently of the original exposure.

C-reactive protein (CRP) levels may fall into this category. Many of the SNPs that could be used to instrument CRP are from upstream inflammatory pathways, while the variant in the promotor region of the *CRP* gene is likely to have a more direct effect on CRP levels themselves (68). If inflammation in general has an influence on the outcome then the *CRP* variant will appear to be an outlier. Estimating the causal influence of CRP on CHD (69,70) is likely to be quite susceptible to this problem, using all 20 variants from Dehghan *et al.* (68) in an IVW estimate suggests a fairly strong protective effect −0.13 (S.E.=0.064), but the *CRP* variant rs2794520 alone gives a much flatter result of 0.009 (S.E.=0.061), consistent with previous analyses (see Appendix 2 for R code on how to obtain these results in MR-Base) (8). By contrast, the (protective) apparent causal influence of CRP on schizophrenia is much more consistent between the *CRP* variant and all other instruments (71), indicating that whether a SNP exhibits horizontal pleiotropy is dependent on the causal question being asked (72,73) (Fig. 1).

Two other outlier removal methods have been used in MR. First, Cook’s distance was used to identify SNPs that exerted a disproportionately large influence on the causal effect in an analysis of body mass index on type 2 diabetes (74).

Second, in Steiger filtering (72), outliers were detected based on the likelihood that they were reverse-causal. Suppose that an analysis is being performed where the hypothesized exposure is actually caused by the hypothesized outcome (i.e. there is a reverse causal relationship). As GWA studies improve in power, the chances of the instruments for the exposure including SNPs that primarily associate with the outcome, and the outcome (or processes leading to the outcome) influencing the apparent exposure, increases. Including those SNPs in the analysis will potentially lead to erroneous inference of causality in the wrong direction. To mitigate this problem Steiger filtering removes those SNPs that explain more of the variance in the outcome than in the exposure. This method could deliver erroneous results under some levels of confounding or reverse causation (42), but it is unlikely to lead to the same problems as the heterogeneity-based outlier removal methods.

## Polygenic Risk Scores

It has been shown consistently that relaxing the significance threshold for GWAS, yielding more associations, can lead to constructed polygenic scores exhibiting better prediction accuracy (75,76). Hence, it is tempting to use a similar strategy in MR because better prediction accuracy of the exposure will improve statistical power (77). There are two potential issues that have received recent attention regarding this approach.

First, as the threshold is relaxed the likelihood of false positive SNP-exposure associations being introduced will increase, which violates the first assumption of MR. A mixture of true and false positive SNPs used as instruments will lead to heterogeneity in the MR analysis. Second, the inclusion of SNPs with smaller genetic effects for the exposure increases the influence of weak instrument bias (69). This is particularly problematic when combined with selection bias (78), where the discovery GWAS is used to estimate the SNP-exposure effects also (i.e. lacking an independent replication). The Mendelian randomization robust adjusted profile score (MR-RAPS) method (55) extends the basic IVW random effects approach by making the weight each variant receives in the analysis a function of the causal effect and the precision of the SNP-exposure association. Under the assumption that pleiotropy is approximately balanced (i.e. it satisfies the InSIDE condition with zero mean, except for a small number of outliers) MR-RAPS enables large numbers of weak instruments well below the conventional GWAS threshold to be included. The new form of weighting utilized by MR-RAPS has also been used to improve the reliability of Cochran’s Q-statistic when testing for heterogeneity due to pleiotropy (51), in particular its false positive (or type I error) rate.

An important question follows from considering the use of many weak instruments, which is a variation of the InSIDE assumption: are the pleiotropic effect distributions monotonic across the range of SNP-exposure effect sizes? The ‘omnigenic’ model of complex traits (69) proposes that almost every gene is related to every phenotype (though whether this is through horizontal or vertical pleiotropy is not clear). Potentially, the SNPs with the smallest effect sizes are those that are most likely to have background effects on all traits. Such a model invites the question of whether improving GWAS sample sizes for SNP discovery, or relaxing the significance threshold, will result in better clarity in MR analyses. An alternative model, and one that is more worrying for MR, is that SNPs with larger effects are the ones more liable to exhibit horizontal pleiotropy, arising because a single variant’s influence on the trait occurs through multiple independent pathways.

## Multivariable Analysis of Several Exposures

In the methods described so far the horizontal pleiotropic effects are detected and adjusted using ‘classical’ univariate statistical techniques (i.e. they may use multiple SNPs but we are modelling a single exposure variable's effect on the outcome). These methods attempt to arrive at unbiased estimates without incorporating additional knowledge of the potential alternative pathways in which SNPs might be operating. But often one can hypothesize what those pathways might be and include them explicitly in the analysis.

Multivariable MR (79–81) attempts to estimate the influence of an exposure on the outcome, conditioning the SNP-exposure effects on their corresponding effects on other putative exposure traits. For example, there is genetic overlap between HDL cholesterol (HDL) and LDL cholesterol (LDL) (82,83). In estimating the influence of LDL on CHD, is it clear that any putative causal effect is not due to the SNPs in fact acting through HDL? If the SNP-CHD effects are proportional to the SNP-LDL effects even after they have been adjusted for the SNP-HDL associations, then this would support the conclusion that LDL has an influence on CHD.

## Negative Controls

An intuitive test of violations of assumptions in MR is to perform the analysis in a context where it is expected that any association under the tested hypothesis is impossible (84–86). This can be performed in different ways. One approach (*negative control outcomes*) is to test if the exposure associates with outcomes that should be impossible, by conducting MR with the instruments that will be used in the focal analysis. If an association is obtained this would indicate that the instruments were in some way invalid (86).

Another approach to negative controls is to test for associations in specific samples where there should be none (*negative control samples*). For example, suppose we want to estimate the influence of alcohol intake on blood pressure. If the instrument for alcohol intake is valid, there should be a SNP-outcome association only among individuals who drink alcohol. However, if there is found to be an association from a sample of individuals who do not drink alcohol then the SNP-outcome association must be arising through a pathway other than the hypothesized exposure, thus proving a violation in at least one of the assumptions (87). Generally, we can view this as a gene-gene (GxG) or gene-environment (GxE) interaction, where the covariate level in which there is no genetic effect is termed the *no relevance point*. It is important to note that grouping by an exogenous variable like sex (88) is safer than potentially endogenous covariates because it avoids the possibility of collider bias (89).

A method termed pleiotropy-robust MR (PRMR) (90) was developed to utilize the no-relevance point to obtain more reliable causal effect estimates. Here, it is assumed that the influence of the SNP on the outcome at the no-relevance point represents the horizontal pleiotropic effect for the rest of the population. The effect estimate from the rest of the population is then adjusted for this pleiotropic effect. This relies on the assumption that the pleiotropic effect is constant across subgroups of the environmental covariate.

In practice there are very few epidemiological examples, including the alcohol example, with a perfect no-relevance point (87,91). MRGxE builds on this approach, by relaxing the requirement that a no-relevance point has to be observed: its value can instead be estimated as long as there is variation in the strength of the SNP-exposure association across subgroups of the environmental covariate (91). While the dependence on GxE or GxG interactions implies that individual level data are required, MRGxE can be performed using summary data if estimates for the SNP-exposure and SNP-outcome associations at different levels of the environmental variable are available. The technique is then analogous to performing MR-Egger regression on the set of covariante stratum-specific SNP-outcome and SNP-exposure association estimates.

## Synthesizing Evidence from Several Models

Interrogating results by analysing how sensitive they are under different assumptions is essential for reliable causal inference. In a *hypothesis-driven analysis* (i.e. a particular exposure is being tested against a particular outcome) a common strategy (92) is to begin with the simplest model, the fixed effects IVW, which has the highest statistical power when all assumptions are met. Sensitivity analyses are then performed that test whether the estimated effect remains consistent using methods that allow different patterns of assumption violations, most notably MR-Egger regression and the median- and mode-based estimators (8). It is also common to see leave-one-out analyses where the causal effect is re-estimated but sequentially omitting a particular instrument each time, to evaluate if any one variant is driving the analysis (8). Extension to systematically leaving out combinations of SNPs is possible also (93).

Sometimes it is the case that it is useful to have a single ‘most likely’ causal effect estimate to select from among the many analyses that have been performed (94). Frameworks for selecting models have been developed recently that attempt to do this.

### Rucker framework

Adapting methodology developed for meta-analysis to the MR context, the Rucker framework (56,95) uses heterogeneity statistics to navigate between different models in a principled manner. One begins by estimating the fixed effects IVW analysis and then calculating Cochran’s Q statistic for heterogeneity. This will indicate whether the SNP-outcome associations are exhibiting inconsistencies which could lead to bias in the fixed effects IVW estimate. If there is substantial heterogeneity then we depart from the fixed effects IVW estimate, moving to a random effect IVW that allows all SNPs to exhibit balanced horizontal pleiotropy.

Next we test for directional pleiotropy—re-estimating the heterogeneity after allowing for a non-zero intercept (using Rucker’s Q’ statistic 96) through a fixed effect MR-Egger analysis. If Q-Q’ is large then this indicates directional horizontal pleiotropy suggesting it more appropriate to use the MR-Egger framework. Finally, if even after accounting for directional pleiotropy Q’ indicates that heterogeneity remains, then ultimately random effects MR-Egger model is selected.

### Model averaging

An alternative to trying to navigate between methods discretely is to average across multiple different models. Thompson *et al.* (2017) (97) applied this idea to MR, using a Bayesian approach to average across three nested models—no pleiotropy (IVW fixed effects), balanced random pleiotropy (IVW random effects) and directional plus random pleiotropy (MR-Egger random effects). Schmidt and Dudbrudge (98) put forward a similar idea in the Bayesian MR-Egger estimator (BMRE), in which prior beliefs about the extent of directional pleiotropy can be used to average between IVW and MR-Egger estimates.

### Mixture of experts

The mixture of experts (MoE) is a machine learning framework in which data can be fed to several different methods (‘experts’), and then the most reliable among them is selected (99). The MR-MoE approach achieves this through *meta learning* (72). First, data simulated under different models of pleiotropy are generated and *summary sets* (Box 1) are produced. Each expert is used to analyse the simulated summary sets. At the same time, characteristics (meta data) about the simulated summary data are generated, e.g. the number of SNPs, sample sizes, heterogeneity, numbers of outliers. Next, a model is fitted that estimates how accurate that expert is for a given summary set based on the summary set’s meta data. Following on, for any given summary set generated from real data, a performance estimate from each expert is made, and the expert predicted to perform the best is selected.

## Towards Coherent Frameworks

A rich and diverse statistical toolkit is emerging that attempts to distil horizontal pleiotropic effects from vertical pleiotropic effects, in order to improve the reliability of causal inference. In Table 2, we outline how the different methods described above can be used in conjunction or in sequence with one another under a range of different scenarios.

Alongside method development, it is now crucial that codebases are maintained in which statistical methods can be deposited and easily applied to arbitrary data. The MR-Base platform integrates an R package with a database, enabling automated causal inference through summary data across a wide range of methods (8). Other software packages are available such as *MendelianRandomization* (96) and *gsmr* (62) and in Stata, *mrrobust* (100). The MR-Base and PhenoScanner (101) databases collate thousands of complete GWAS summary datasets, and coverage of human traits with well powered GWAS summary data will continue to grow. For most of the methods described in this review, the horizontal pleiotropic effects are modelled using knowledge only of the SNP effects on the exposure and the outcome. But when massive amounts of data are available, we are now presented with opportunities to attempt to model the pleiotropic relationships explicitly. The MR-EvE graph database (MR of ‘Everything versus Everything’) goes one step towards this goal (72). The next major transformation in MR is likely to involve the improvement of causal inference by incorporating information from beyond the SNP-exposure and SNP-outcome effects, in the spirit of triangulation of evidence (16,18).

## Figures and Tables

**Appendix figure 1.**
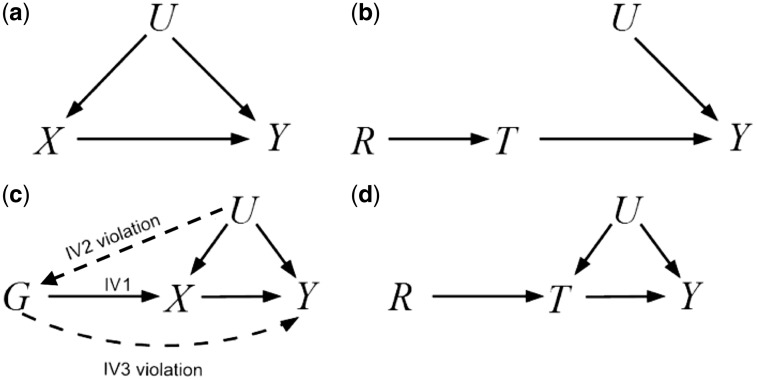
**(A)** Unobserved confounding (U) makes it impossible to be fully confident that an association between risk factor X and outcome Y represents a measure of causal effect of X on Y. **(B)** In a perfect RCT, randomization to treatment (T) removes the possibility of confounding, enabling the causal effect of T on Y to be estimated. **(C)** MR uses genetic variants (G) that explain some variation in the exposure X to estimate the causal effect of X on Y. G must satisfy the instrumental variable assumptions, encoded by the solid arrows (and the strict absence of the dotted arrows) in (C). **(D)** Instrumental variable methods can also be used in clinical trials when randomization is imperfect because some patients do not receive the treatment they were originally assigned. This is referred to as `non-compliance’. An MR analysis is conceptually and mathematically equivalent to the analysis of RCT data in the presence of non-compliance, where the SNP (G) and exposure (X) proxy for randomization (R) and treatment (T), respectively.

## Appendix 1. The MR framework and its assumptions

Mendelian randomization (MR) is a special case of instrumental variable (IV) analysis in which genetic factors are used to proxy the exposure variable, because unlike the exposure variable the genetic factor is less liable to reverse causation or confounding. Suppose that a SNP is known to influence the exposure of interest (IV assumption 1). Whether an individual inherits the exposure-increasing or -decreasing allele is a random process, analogous to lifetime random assignment to a treatment or control group in an RCT. Unlike in a perfectly conducted RCT, in which assignment to treatment group perfectly predicts whether the treatment is taken or not (Appendix figure 1), a genetic factor usually exerts only a very small effect on the exposure. Many other variables will influence the value of the exposure, and if they also influence the outcome, they would `confound’ the exposure-outcome relationship (as represented by the variable U in Appendix figure 1). As long as the SNP is a valid IV, MR can return unbiased estimates for the causal effect of the exposure on the outcome in the presence of such confounding. MR can therefore be viewed as an RCT with non-compliance, as illustrated in Appendix figure 1.

If the SNP associates with the outcome then one can qualitatively conclude that the exposure causes the outcome, in the same way that the analysis of trial data according to the intention to treat (ITT) principle also provides a valid test for a non-zero treatment effect. An MR analysis goes further by providing a quantitative estimate of the causal effect. The validity of both of these conclusions depend on two further core assumptions—that the SNP does not associate with confounders (IV assumption 2), and does not influence the outcome through some pathway other than the exposure (IV assumption 3). Assumption 1 is easy to prove through performing genome wide association studies and replicating strong signals in independent studies, but assumptions 2 and 3 are impossible to prove.

The most popular method over the last few years to perform MR is the two-sample summary data case (2SMR), where the jth SNP-exposure effect estimate ${\hat{\gamma }}_{j}$ and its variance $var(\varepsilon_{x_{j}})$ are obtained from a published GWAS, and the corresponding SNP-outcome effect, $\hat{\Gamma_{j}}$ and variance $var(\varepsilon_{y_{j}})$ is obtained from another sample. A simple formulation of the factors that influence the SNP effect estimates for the exposure and the outcome are as follows:

$${\hat{\gamma }}_{j}=\gamma_{j}+k_{x}\psi_{j}+\varepsilon_{x_{j}}$$

$$\hat{\Gamma_{j}}=\alpha_{j}+k_{y}\psi_{j}+\beta {(\gamma }_{j}+k_{x}\psi_{j})+\varepsilon_{y_{j}}$$

where β is the causal effect of *x* on *y*, and $\alpha_{j}$ represents the jth SNP’s horizontal pleiotropic effect (Box 3). A confounder influencing x and y with effects κ_x_ and κ_y_, respectively, could also have effects on the SNP with effect ψ_j_. When $\alpha_{j}=0$ and the SNP does not associate with confounders the Wald ratio, $\hat{\beta_{j}}=\hat{{\;\Gamma }_{j}}/{\hat{\gamma }}_{j}$ is a causal effect estimate based on the jth SNP. When there are multiple SNPs available as instruments the Wald ratios from each SNP are meta-analysed to give an overall estimate. Using this framework, Bowden *et al.* (59) outlined the key set of assumptions for the 2SMR case (Table 1) that relate to the parameters in the above equations.

## Appendix 2

R code to produce MR estimates of CRP on coronary heart disease and schizophrenia

library(TwoSampleMR)

library(MRInstruments)

library(dplyr)

data(gwas_catalog)

# Get the instruments for CRP

crp <- subset(gwas_catalog, grepl(“C-reactive protein”, Phenotype) & grepl(“Dehghan”, Author)) %>% format_data

# Perform MR of CRP on coronary heart disease

chd <- extract_outcome_data(crp$SNP, 7)

d <- harmonise_data(crp, chd)

mr(d)

# Perform using only the CRP variant

mr(subset(d,SNP == subset(crp, gene.exposure==“CRP”)$SNP))

# Perform MR of CRP on schizophrenia

scz <- extract_outcome_data(crp$SNP, 22)

d <- harmonise_data(crp, scz)

mr(d)

# Perform using only the CRP variant

mr(subset(d,SNP == subset(crp, gene.exposure==“CRP”)$SNP))
